# Silicon Nanoparticles Alter Soybean Physiology and Improve Nitrogen Fixation Potential Under Atmospheric Carbon Dioxide (CO_2_)

**DOI:** 10.3390/plants14132009

**Published:** 2025-06-30

**Authors:** Jingbo Tong

**Affiliations:** School of Water Conservancy and Civil Engineering, Northeast Agricultural University, Harbin 150038, China; tongjingbo@neau.edu.cn

**Keywords:** elevated CO_2_, soybean, photosynthesis, nitrogen fixing potential

## Abstract

The interactive effects between nano-silicon dioxide (*n*-SiO_2_) and elevated CO_2_ (eCO_2_; 645 ppm) on soybean physiology, nitrogen fixation, and nutrient dynamics under climate stress remain underexplored. This study elucidates their combined effects under ambient (aCO_2_; 410 ppm) and eCO_2_ conditions. eCO_2_ + *n*-SiO_2_ synergistically enhanced shoot length (30%), total chlorophyll (112.15%), and photosynthetic rate (103.23%), alongside improved stomatal conductance and intercellular CO_2_ (17.19%), optimizing carbon assimilation. Nodulation efficiency increased, with nodule number and biomass rising by 48.3% and 53.6%, respectively, under eCO_2_ + *n*-SiO_2_ versus aCO_2_. N-assimilation enzymes (nitrate reductase, nitrite reductase, glutamine synthetase, glutamate synthase) surged by 38.5–52.1%, enhancing nitrogen metabolism. Concurrently, phytohormones (16–21%) and antioxidant activities (15–22%) increased, reducing oxidative markers (18–22%), and bolstering stress resilience. Nutrient homeostasis improved, with P, K, Mg, Cu, Fe, Zn, and Mn elevating in roots (13–41%) and shoots (13–17%), except shoot Fe and Zn. These findings demonstrate that *n*-SiO_2_ potentiates eCO_2_-driven benefits, amplifying photosynthetic efficiency, nitrogen fixation, and stress adaptation through enhanced biochemical and nutrient regulation. This synergy underscores *n*-SiO_2_ role in optimizing crop performance under future CO_2_-rich climates, advocating nano-fertilizers as sustainable tools for climate-resilient agriculture.

## 1. Introduction

In soybean, elevated CO_2_ can stimulate growth by increasing photosynthetic rates, but it also induces physiological and biochemical changes that may negatively impact seed quality. Research indicates that while carbohydrate accumulation may increase under elevated CO_2_, nitrogen and mineral concentrations tend to decline due to reduced transpiration and altered nutrient uptake [1,2]. Additionally, interactions between elevated CO_2_ and other environmental stressors, such as drought, can exacerbate these effects, leading to fluctuations in seed composition, including protein and fatty acid profiles [3,4]. Given these challenges, innovative strategies are required to balance the benefits of CO_2_ fertilization with the need to maintain nutrient integrity in crops.

Nanotechnology has emerged as a transformative tool to enhance crop resilience under climatic stressors [5]. Previous studies have demonstrated the potential of nanomaterials (NMs) in enhancing plant responses under elevated CO_2_ conditions. For instance, chitosan nanoparticles have been shown to upregulate carbon and nitrogen metabolism in soybean [6], while foliar application of CeO_2_ nanoparticles improved growth and nutritional quality in *Spinacia oleracea* L. [7], highlighting their role in promoting food security under future climatic conditions. Similarly, Se-NMs enhanced nutrient concentrations and biomass in wheat, demonstrating their potential to optimize growth conditions under increased CO_2_ levels [8]. Similarly, Silicon nanoparticles (*n*-SiO_2_), in particular, exhibit unique physicochemical properties that improve plant water-use efficiency, nutrient uptake, and stress tolerance [9]. Silicon, though non-essential for most plants, amplifies defense mechanisms against biotic and abiotic stresses by reinforcing cell walls, modulating antioxidant systems, and enhancing photosynthetic efficiency [10]. In legumes, silicon supplementation strengthens root architecture, promotes nodulation, and stabilizes nitrogenase activity under drought and salinity [11]. However, the interplay between *n*-SiO_2_ and eCO_2_ in soybean remains unexplored, despite the potential for synergistic effects on carbon assimilation and nitrogen dynamics.

In the current work, soybean plants were employed as the model leguminous species to thoroughly investigate how soybean responds to *n*-SiO_2_ exposure under elevated CO_2_. The detailed studies on soybean plants are particularly necessary because soybean (*Glycine max*) serves as a vital crop for food, animal feed, and biofuel, contributing significantly to global legume production. Its symbiotic relationship with N_2_-fixing bacteria enables soybeans to fix approximately 16.4 million metric tons of nitrogen annually, significantly reducing reliance on synthetic nitrogenous fertilizers. This study examines how *n*-SiO_2_ influences soybean physiology and nitrogen fixation under elevated CO_2_, focusing on four key aspects: (1) physiological adaptation, assessing *n*-SiO_2_ role in enhancing mesophyll conductance and chloroplast stability; (2) net photosynthetic activity, evaluating its impact on Rubisco activity and electron transport efficiency; (3) root and nodule health, investigating how *n*-SiO_2_ stabilizes nodule function amid shifting C:N ratios; and (4) biochemical and nitrogen metabolism, exploring its role in mitigating oxidative stress and optimizing nitrogen assimilation. By addressing these dimensions, this research provides critical insights into *n*-SiO_2_ potential to enhance soybean resilience and productivity in a CO_2_-enriched environment.

## 2. Results

### 2.1. n-SiO_2_ Alters Soybean Physiology Under Elevated CO_2_

**Figure 1:**
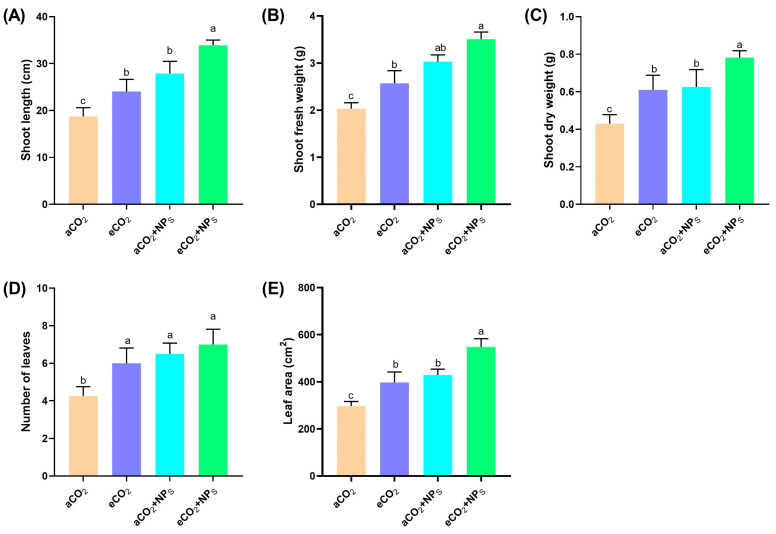
The effects of *n*-SiO_2_ on soybean physiological traits under ambient (aCO_2_) and elevated (eCO_2_) CO_2_ conditions. Data are presented for (**A**) shoot length, (**B**) shoot fresh weight, (**C**) shoot dry weight, (**D**) number of leaves, and (**E**) leaf area. Soybean plants exposed to elevated CO_2_ showed significant improvements in all measured traits compared to ambient CO_2_, with *n*-SiO_2_ supplementation further enhancing these parameters. Different letters above the bars indicate significant differences (*p* < 0.05) between treatments.

The increasing atmospheric CO_2_ levels due to climate change pose significant challenges to agricultural systems, particularly in enhancing crop growth and nutritional quality. The current study aimed to investigate how *n*-SiO_2_ influences soybean physiology, including shoot growth, biomass accumulation, leaf development, and photosynthetic efficiency, under ambient (aCO_2_) and elevated CO_2_ levels (Figure 1). The results indicate that eCO_2_ alone significantly enhanced soybean growth, with a 30% increase in shoot length and a 58% increase in shoot fresh weight compared to aCO_2_ conditions. These findings align with previous reports that elevated CO_2_ improves photosynthesis and biomass accumulation in C3 plants like soybean [12]. However, the addition of *n*-SiO_2_ under eCO_2_ further boosted shoot length by an additional 38%, indicating that *n*-SiO_2_ may enhance cell elongation and chloroplast stability, optimizing photosynthetic efficiency [13]. Similarly, shoot fresh weight increased by an additional 11% with *n*-SiO_2_, reflecting its role in improving nutrient uptake and water-use efficiency, which are critical for maximizing plant productivity under stress. These results are consistent with studies showing that *n*-SiO_2_ can promote biomass accumulation through enhanced nutrient acquisition and improved physiological responses to environmental stress [14,15].

Shoot dry weight followed a similar pattern, increasing by 38% under eCO_2_ and by an additional 28% with *n*-SiO_2_ supplementation (Figure 1). This suggests that *n*-SiO_2_ helps in improving the plant’s metabolic activity and stress tolerance, thereby enhancing its ability to accumulate dry matter under elevated CO_2_ conditions [16]. Moreover, leaf number increased by approximately 27% with *n*-SiO_2_ supplementation, likely due to enhanced auxin signaling and root function [17]. These changes in leaf morphology are crucial for optimizing the plant’s ability to capture light and perform photosynthesis effectively. The leaf area, a critical factor for photosynthetic capacity, increased by 53% under eCO_2_, with an additional 50% increase observed when *n*-SiO_2_ was applied (Figure 1). These results suggest that *n*-SiO_2_ enhances the plant’s capacity for light capture and carbon fixation, improving overall growth under elevated CO_2_ conditions. The significant increase in leaf area aligns with previous findings that *n*-SiO_2_ can enhance photosynthetic efficiency by improving leaf expansion and reducing oxidative stress [14,15]. A recent study reported that nanoparticles (CeO_2_-NPs) significantly increased plant physiological growth indicators (shoot and root length, shoot and root weight) by 40.5% to 84.6% at 100 ppm under the eCO_2_ conditions [7]. Another study documented that chitosan nanoparticles (CS-NPs) significantly enhanced soybean shoot and root biomass by ~1.6-fold under elevated CO_2_ conditions compared to the control group [6]. SiO_2_-NPS significantly increased (36%) the cherry radish fresh weight [18]. Regarding the soybean growth, ionic Si significantly increased the shoot dry weight by 23% compared to the control group [19]. These results collectively highlight that *n*-SiO_2_ plays a crucial role in optimizing soybean growth under elevated CO_2_ conditions. The observed enhancements in shoot elongation, biomass accumulation, leaf development, and photosynthetic potential demonstrate the potential of *n*-SiO_2_ to mitigate the negative effects of elevated CO_2_ and improve soybean productivity. This aligns with the growing body of literature emphasizing the role of nanomaterials, like *n*-SiO_2_, in enhancing crop resilience and performance under future climate scenarios with higher CO_2_ levels [6,7].

### 2.2. Enhancement of Photosynthesis Activity in Soybean Under Elevated CO_2_ Induced by n-SiO_2_

**Figure 2:**
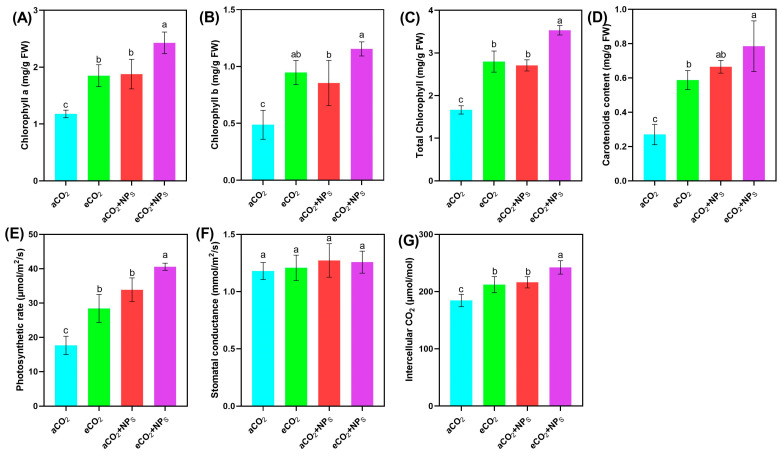
Effects of *n*-SiO_2_ on soybean photosynthetic parameters under ambient (aCO_2_) and elevated (eCO_2_) CO_2_ conditions. Data represent (**A**) chlorophyll *a*, (**B**) chlorophyll *b*, (**C**) total chlorophyll, (**D**) carotenoid content, (**E**) photosynthetic rate, (**F**) stomatal conductance, and (**G**) intercellular CO_2_ concentration. Different letters above the bars indicate significant differences (*p* < 0.05) between treatments.

The application of *n*-SiO_2_ significantly enhanced soybean photosynthetic performance under eCO_2_ conditions, demonstrating their potential to optimize plant physiology in response to climate change. Under eCO_2_ (645 ppm), *n*-SiO_2_ treatment led to marked improvements in key photosynthetic parameters compared to aCO_2_, highlighting a strong synergistic effect between elevated CO_2_ and *n*-SiO_2_. A substantial increase in chlorophyll content was observed, with chlorophyll *a* and *b* rising by 106.16% and 136.92%, respectively, and a 112.15% increase in total chlorophyll (Figure 2). These results align with previous studies [6,7] and suggest enhanced chlorophyll biosynthesis and stabilization of the photosynthetic apparatus, likely due to more efficient nutrient uptake, enhanced nitrogen metabolism, and reduced oxidative stress. These changes facilitate greater light absorption and energy conversion, which are critical for maintaining high photosynthetic activity under eCO_2_. Further supporting this, Ahsan et al. documented that *n*-SiO_2_ nanoparticles penetrate chloroplasts, reaching photosystem II reaction centers, stimulating electron transmission and light absorption, and thus enhancing photosynthetic efficacy. This mechanism contributes to the elevated chlorophyll concentration, leading to improved photosynthesis and overall plant performance [20].

Additionally, carotenoid content, crucial for light harvesting and photoprotection, increased by 41.96% under eCO_2_ with *n*-SiO_2_ treatment. This suggests strengthened antioxidant defenses, mitigating photooxidative stress commonly associated with high CO_2_ levels and intense light exposure. The elevated carotenoid levels also stabilize chlorophyll molecules, supporting sustained photosynthetic efficiency. In parallel, photosynthetic efficiency, as indicated by the net photosynthetic rate (Pn), stomatal conductance (Gs), and intercellular CO_2_ concentration (Ci), showed significant improvements. Previous studies have documented that elevated CO_2_ initially boosts Pn but can lead to down-regulation over time, accompanied by reduced Gs and increased Ci [21,22]. Another study reported that nanoparticles (CeO_2_-NPs) significantly enhanced photosynthetic indicators (chlorophyll *a*, *b*, and total chlorophyll content) by 85.3% to 398.8% at 50–100 mg L^−1^ (CeO_2_-NPs) under the elevated CO_2_ conditions [7]. Additionally, CS-NPs at 40–65 μg mL^−1^ improved the soybean photosynthetic activities by 1.5 to 2.9 folds more than that ambient CO_2_ group [6]. SiO_2_-NPs, as a nano-fertilizer, increased the plant’s (cherry radish) total chlorophyll and carotenoids contents by 14.2–18.7% [18]. Wang et al. comparing the potential of silicate and SiO_2_-NPs, observed that SiO_2_-NPs significantly improved soybean photosynthesis related indicators compared to both the control and silicate groups [13]. In our study, the photosynthetic rate increased by 103.23% under eCO_2_ + *n*-SiO_2_, reflecting enhanced carbon assimilation. This was supported by improved stomatal conductance, which facilitated more efficient CO_2_ influx, and a 17.19% rise in intercellular CO_2_ concentration, indicating better CO_2_ diffusion and utilization within the leaf tissues. These physiological changes suggest that *n*-SiO_2_ helps optimize gas exchange processes, thereby enhancing photosynthetic performance and overall plant productivity [23]. This is consistent with previous reports that nanomaterials can enhance Pn, counteract the reduction of Gs, and improve overall photosynthetic performance under elevated CO_2_ [6,7]. Overall, the integration of *n*-SiO_2_ with elevated CO_2_ conditions significantly boosts soybean growth by enhancing chlorophyll synthesis, optimizing gas exchange, and reinforcing photoprotective mechanisms. These findings underscore the potential of *n*-SiO_2_ as an effective strategy to improve crop performance and resilience in the face of rising atmospheric CO_2_ concentrations.

### 2.3. Synergistic Effects of Silicon Nanoparticles on Root Morphology and in Soybean Under Elevated CO_2_

**Figure 3:**
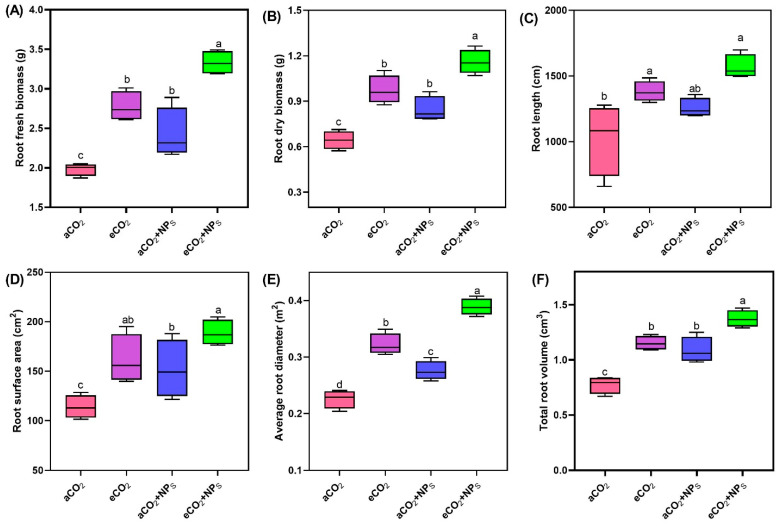
Effects of SiO_2_-NPs under aCO_2_ and eCO_2_ conditions on root physiological traits of soybean. (**A**) Root fresh biomass, (**B**) root dry biomass, (**C**) root length, (**D**) root surface area, (**E**) average root diameter, and (**F**) total root volume in soybean plants exposed to different CO_2_ concentrations and SiO_2_-NPs treatments. Box plots represent the median (line), interquartile range (box), and minimum/maximum values (whiskers). Different letters indicate significant differences among treatments (*p* < 0.05).

The application of *n*-SiO_2_ under eCO_2_ significantly improved various root physiology indicators in soybean plants. Root fresh biomass (Figure 3A) showed a clear enhancement with a 48.3% upregulation under eCO_2_ + *n*-SiO_2_ compared to aCO_2_, with values increasing from 2.3 g to 3.5 g. This increase suggests that *n*-SiO_2_ plays a role in improving overall root growth and biomass accumulation under eCO_2_ conditions. Similarly, root dry biomass (Figure 3B) increased by 43.3% under eCO_2_ + *n*-SiO_2_, further confirming the positive impact of *n*-SiO_2_ on root system development under stress conditions. Root length (Figure 3C) followed a similar trend, with a 38.5% increase under eCO_2_ + *n*-SiO_2_ compared to aCO_2_. This finding is consistent with enhanced root elongation and expansion, which is crucial for optimizing nutrient and water uptake in challenging environmental conditions. The improvement in root length may be attributed to the combined effects of elevated CO_2_ and *n*-SiO_2_, which likely enhance root cell division and elongation processes. Root surface area (Figure 3D) increased by 28.5% under eCO_2_ + *n*-SiO_2_, further supporting the notion that *n*-SiO_2_ facilitates greater root surface area, which is vital for improving nutrient and water absorption. The average root diameter (Figure 3E) also showed a 13.5% increase under eCO_2_ + *n*-SiO_2_, indicating that *n*-SiO_2_ not only boosts root surface area but also enhances root structure and thickness, contributing to a more robust root system. Total root volume (Figure 3F) exhibited a 23.4% increase under eCO_2_ + *n*-SiO_2_, reinforcing the overall improvements in root morphology and physiology. The increase in root volume is likely to enhance the plant’s ability to absorb water and nutrients [24,25], which is critical for maintaining plant growth under elevated CO_2_ conditions [26].

The observed enhancement of root physiology in soybean plants under eCO_2_ induced by *n*-SiO_2_ can be attributed to several synergistic effects. Elevated CO_2_ typically stimulates plant growth by enhancing photosynthesis, leading to increased carbon allocation to roots [22,27,28]. Mukarram et al. investigated the impact of SiNPs on crop health through their direct influence on various biochemical and physiological processes, such as increased photosynthetic activity, enhanced nutrient uptake, improved nitrogen metabolism, and elevated enzymatic activity, all of which directly enhanced plant growth and yield [29]. Another recent study documented that *n-*SiO_2_ at 100 mg kg^−1^ significantly increased maize root weight, length, and number of roots by 4.5% to 20% compared to a control group [30]. The addition of *n*-SiO_2_ further amplifies this effect by improving root architecture, increasing root biomass, surface area, and volume. The improvement in root growth can be attributed to enhanced photosynthesis, which facilitates belowground carbon sequestration [31]. Larger root systems with optimized architecture are also expected to improve water and nutrient acquisition by plants, as well as indirectly stimulate photosynthetic CO_2_ capture. These findings are consistent with our study, where the enhancement of root physiological traits was evident under eCO_2_ + *n*-SiO_2_ treatment.

The increase in root diameter and surface area suggests that *n*-SiO_2_ promotes root cell expansion and tissue development, improving the plant’s ability to absorb nutrients and water more efficiently. Such changes are crucial for optimizing plant growth under water-limited conditions and improving stress resilience. Previous studies have highlighted that *n*-SiO_2_ promotes secondary and lateral root development, further improving nutrient uptake and overall root function [11,20,32]. This supports our observation that *n*-SiO_2_ enhances root structure and function, making the plant more resilient under challenging environmental conditions. Overall, these results underscore the potential of *n*-SiO_2_ to optimize plant growth and nutrient uptake under elevated CO_2_, making it a promising tool for enhancing crop resilience and ensuring food security in the face of climate change. The improvement in root physiology and nitrogen metabolism demonstrates that *n*-SiO_2_ could be a key strategy in enhancing crop productivity, particularly under elevated CO_2_ conditions.

### 2.4. Modulation in Biochemical Response in Soybean Under n-SiO_2_-Induced by Elevated CO_2_

**Figure 4:**
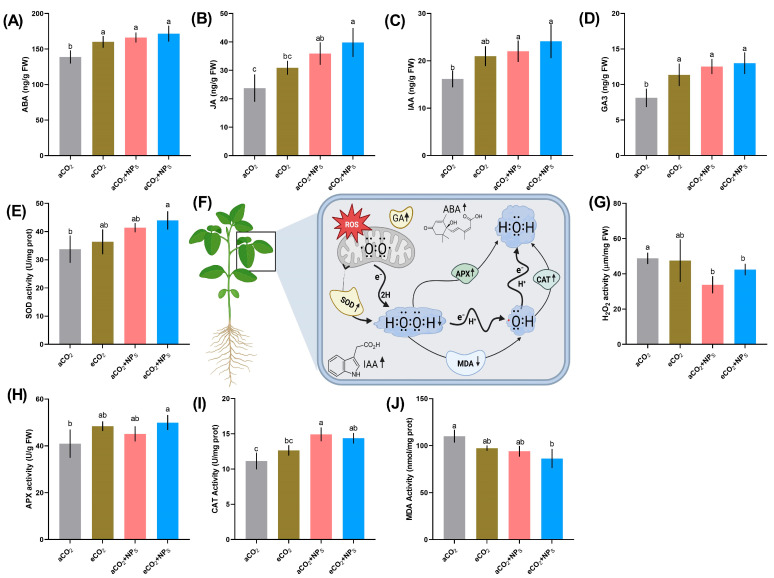
Effects of elevated CO_2_ and nanoparticle treatments on phytohormone levels and antioxidant enzyme activities in plants. (**A**–**D**) Changes in abscisic acid (ABA), jasmonic acid (JA), indole-3-acetic acid (IAA), and gibberellic acid (GA_3_) levels under ambient CO_2_ (aCO_2_), elevated CO_2_ (eCO_2_), eCO_2_ with nanoparticles (eCO_2_ + NPs), and aCO_2_ with nanoparticles (aCO_2_ + NPs). (**E**–**J**) Antioxidant enzyme activities, including superoxide dismutase (SOD), hydrogen peroxide (H_2_O_2_), ascorbate peroxidase (APX), catalase (CAT), and malondialdehyde (MDA), under the same treatments. (**F**) Schematic representation of the antioxidant defense system in plants, illustrating the role of reactive oxygen species (ROS), enzymatic detoxification pathways (SOD, APX, and CAT), and interactions with phytohormones. Different letters indicate statistically significant differences among treatments (*p* < 0.05). Error bars represent standard deviations (SD).

The combined application of *n*-SiO_2_ and eCO_2_ significantly influenced key phytohormones that regulate plant growth, stress adaptation, and development. Abscisic acid (ABA) content (Figure 4A) increased by 16.5% under eCO_2_ + *n*-SiO_2_ compared to aCO_2_, indicating enhanced plant resilience through improved stomatal regulation. ABA, which plays a crucial role in mitigating water loss under elevated CO_2_, regulates stomatal closure, and the addition of *n*-SiO_2_ further strengthens this effect by improving water retention. Similarly, jasmonic acid (JA) levels (Figure 4B) showed a 35.2% increase under eCO_2_ + *n*-SiO_2_, suggesting a reinforced defense response. JA is involved in systemic resistance and stress signaling, and its enhanced levels indicate a stronger adaptive response to oxidative stress. Indole-3-acetic acid (IAA) content (Figure 4C) increased by 21.7% under eCO_2_ + *n*-SiO_2_, signifying improved root and shoot development. As a primary auxin responsible for cell elongation and differentiation, the upregulation of IAA suggests that *n*-SiO_2_ enhances cell expansion, optimizing root architecture for better nutrient and water uptake under eCO_2_. Likewise, gibberellic acid (GA3) levels (Figure 4D) increased by 18.9%, indicating enhanced shoot elongation and biomass accumulation. GA3, essential for stem elongation and seed germination, likely promotes growth pathways under eCO_2_, supporting improved plant performance under stress conditions. The enhanced phytohormone levels observed in soybean under eCO_2_ and *n*-SiO_2_ treatments suggest a synergistic mechanism that could improve plant resilience and productivity. Previous studies have shown that nanoparticles like chitosan can induce a similar phytohormone accumulation under elevated CO_2_, which aligns with our findings [6]. Phytohormones, particularly auxins (IAA) and gibberellins (GA), play pivotal roles in regulating plant physiological and biochemical processes, including photosynthesis [33]. These hormones are synthesized within chloroplasts and influence chloroplast development, size, thylakoid membrane organization, and chlorophyll content, ultimately determining photosynthetic capacity [33]. Furthermore, phytohormones like IAA and GA are involved in a crosstalk with sugar metabolism, linking growth regulation and energy production [34,35]. Similar to our findings, elevated CO_2_ has been shown to increase phytohormone levels in *Arabidopsis thaliana* and soybean, reflecting biochemical adjustments to enhanced CO_2_ availability [36]. These observations confirm that improvements in IAA and GA levels under eCO_2_ and *n*-SiO_2_ treatments help enhance photosynthesis and overall growth, highlighting the potential of *n*-SiO_2_ to boost plant physiological functions under changing environmental conditions.

The antioxidant defense system plays a crucial role in mitigating oxidative stress caused by reactive oxygen species (ROS) [37]. Our study demonstrates that the application of *n*-SiO_2_ under elevated CO_2_ (eCO_2_) significantly enhanced antioxidant enzyme activities, thereby improving the plant’s ability to counteract oxidative stress. Superoxide dismutase (SOD) activity (Figure 4E) increased by 19.4% under eCO_2_ + *n*-SiO_2_ compared to aCO_2_, highlighting an improved ROS scavenging capacity. Since SOD is the first line of defense against oxidative stress by converting superoxide radicals into hydrogen peroxide (H_2_O_2_) [38], its enhanced activity suggests that *n*-SiO_2_ helps reduce ROS accumulation, preventing cellular damage and maintaining redox homeostasis. As a consequence of increased SOD activity, H_2_O_2_ content (Figure 4G) decreased by 22.1% under eCO_2_ + *n*-SiO_2_, confirming the effective mitigation of oxidative stress. Lower H_2_O_2_ levels indicate that the antioxidative system efficiently neutralized excess ROS, thereby reducing oxidative damage to cellular structures [39]. To further strengthen ROS detoxification, ascorbate peroxidase (APX) activity (Figure 4H) exhibited a 15.8% increase. APX plays a critical role in converting H_2_O_2_ into water, preventing its accumulation and minimizing oxidative damage. Similarly, catalase (CAT) activity (Figure 4I) showed a 23.7% increase, reinforcing the plant’s ability to decompose excess H_2_O_2_ into water and oxygen. Higher CAT activity further supports the role of *n*-SiO_2_ in enhancing antioxidative defense, allowing plants to better cope with oxidative stress under eCO_2_ conditions. Additionally, malondialdehyde (MDA) content (Figure 4J) decreased by 18.6%, demonstrating lower lipid peroxidation and improved membrane stability. Since MDA is a key indicator of oxidative membrane damage, its reduction suggests that *n*-SiO_2_ contributes to protecting plant cells from oxidative stress by maintaining membrane integrity. While *n*-SiO_2_ application significantly enhanced the antioxidant defense response, the effects of cerium oxide nanoparticles (CeO_2_ NPs) under eCO_2_ exhibited a different trend. The exposure of plants to CeO_2_ NPs under eCO_2_ conditions did not induce oxidative stress, as evidenced by stable MDA and H_2_O_2_ levels. Similarly, no oxidative stress was observed under CeO_2_ NPs treatment at any concentration under both ambient CO_2_ (aCO_2_) and eCO_2_ conditions [7]. This suggests that, unlike some metal-based nanoparticles that induce ROS accumulation, CeO_2_ NPs do not generate excessive ROS, maintaining cellular homeostasis. Despite the absence of oxidative stress, CeO_2_ NPs altered antioxidant enzyme activities, potentially enhancing plant stress resilience [7]. At low concentrations, CeO_2_ NPs positively impacted the overall antioxidant capacity of plants, likely due to their enzyme-mimicking properties. CeO_2_ NPs exhibit both oxidant and antioxidant behavior in plants, particularly by mimicking superoxide dismutase (SOD) activity, which enhances plant tolerance to abiotic stress. However, previous studies have reported mixed responses regarding the effect of CeO_2_ NPs on antioxidant enzymes. In spinach plants, no significant change in SOD activity was observed across all CeO_2_ NPs treatments under both aCO_2_ and eCO_2_ conditions. In contrast, CAT activity increased under both CO_2_ conditions, while APX activity exhibited a significant upregulation at 100 mg/L CeO_2_ NPs under eCO_2_ [7]. These findings suggest that the effects of CeO_2_ NPs on antioxidant enzyme activity are enzyme specific and influenced by environmental conditions. Furthermore, contradictory findings have been reported concerning oxidative stress markers. Some studies indicate that higher concentrations of NMs lead to increased MDA content and ion membrane leakage, while others found no significant oxidative damage. These discrepancies could be attributed to variations in plant growth conditions, species-specific responses, application methods, and differences in concentration and duration of exposure.

### 2.5. n-SiO_2_ Enhances Nodule Health and Biological Nitrogen Fixation Potential in Soybean Under Elevated CO_2_

**Figure 5:**
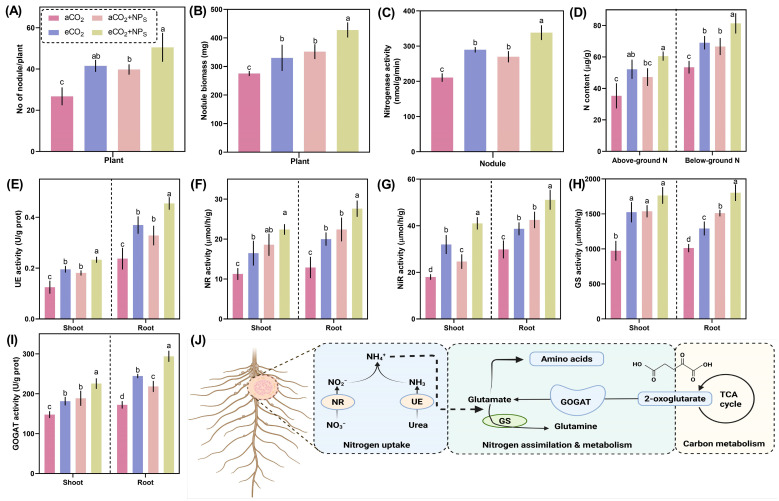
Effects of *n*-SiO_2_ on soybean root and nodule physiological parameters under ambient (aCO_2_) and elevated (eCO_2_) CO_2_ conditions. Data represent (**A**) number of nodules per plant, (**B**) nodule biomass, (**C**) nitrogenase activity, (**D**) nitrogen content in above- and below-ground tissues, (**E**) Urease (UE) activity, (**F**) nitrate reductase (NR) activity, (**G**) nitrite reductase (NIR) activity, (**H**) glutamine synthetase (GS) activity, and (**I**) glutamate synthase (GOGAT) activity in soybean shoots and roots. (**J**) Schematic representation of nitrogen uptake, assimilation, and metabolism pathways in response to *n*-SiO_2_ and elevated CO_2_. Different letters indicate significant differences (*p* < 0.05) among treatments.

The impact of eCO_2_ on nodulation and biological nitrogen fixation in soybean, particularly when influenced by *n*-SiO_2_, has not been thoroughly investigated. Our study shows that the application of *n*-SiO_2_ under eCO_2_ significantly enhances nodule health and nitrogen fixation potential in soybean plants compared to aCO_2_ conditions. The number of nodules per plant (Figure 5A) significantly increased under eCO_2_ + *n*-SiO_2_, showing a 48.3% upregulation compared to aCO_2_ and a 22.5% increase relative to eCO_2_ alone. Similarly, nodule biomass (Figure 5B) was enhanced by 53.6% under eCO_2_ + *n*-SiO_2_, suggesting that *n*-SiO_2_ supplementation amplifies the beneficial effects of eCO_2_ on nodulation efficiency. Previous studies have documented the positive influence of eCO_2_ on root physiology, including increased root nodule growth, nutrient uptake efficiency, and enhanced water absorption capacity [22,27,28,40]. Our findings indicate that *n*-SiO_2_ further strengthens these effects, potentially by promoting secondary and lateral root development, leading to improved overall nutrient acquisition and stress resilience [11,20,32].

Nitrogenase activity (Figure 5C), a key determinant of biological nitrogen fixation, was significantly enhanced in response to *n*-SiO_2_ supplementation. Compared to aCO_2_, nitrogenase activity exhibited a 65.2% increase under eCO_2_ + *n*-SiO_2_, confirming that *n*-SiO_2_ plays a crucial role in enhancing nitrogen fixation efficiency. Likewise, total nitrogen content (Figure 5D) in both above-ground and below-ground tissues increased markedly, with a 34.7% rise in above-ground nitrogen and a 41.2% increase in below-ground nitrogen under eCO_2_ + *n*-SiO_2_ compared to aCO_2_. These results suggest that *n*-SiO_2_ enhances nitrogen assimilation by facilitating nitrogen uptake and transport throughout the plant.

The impact of *n*-SiO_2_ on key nitrogen metabolism enzymes was also evident. Urease (UE) activity (Figure 5E), which plays a critical role in nitrogen remobilization, increased by 43.8% in root nodule tissues under eCO_2_ + *n*-SiO_2_. Similarly, nitrate reductase (NR) and nitrite reductase (NIR) activities (Figure 5F,G) exhibited significant upregulation, increasing by 38.5% and 52.1%, respectively, under eCO_2_ + *n*-SiO_2_ compared to aCO_2_. These enhancements indicate an improved nitrate assimilation pathway, leading to more efficient nitrogen utilization. Furthermore, glutamine synthetase (GS) and glutamate synthase (GOGAT) activities (Figure 5H,I), both essential for nitrogen assimilation, were significantly enhanced under eCO_2_ + *n*-SiO_2_. GS activity increased by 47.2%, while GOGAT activity exhibited a 58.3% upregulation, reinforcing the improved nitrogen metabolism and assimilation associated with *n*-SiO_2_ supplementation under eCO_2_ conditions. The schematic representation (Figure 5J) provides an overview of nitrogen uptake and assimilation pathways, highlighting the mechanisms through which *n*-SiO_2_ improves nitrogen fixation and metabolic assimilation under eCO_2_. Several studies have reported that increased CO_2_ levels can stimulate nitrogen fixation, potentially due to enhanced photosynthetic rates and improved root architecture [41] Additionally, root exudate composition under eCO_2_ can influence microbial communities in the rhizosphere, with beneficial rhizobacteria playing a crucial role in promoting root nodule health and nitrogen fixation efficiency [42]. Given that eCO_2_ typically enhances nodule biomass and nitrogen fixation in legumes like soybean, our results suggest that *n*-SiO_2_ further strengthens this effect by optimizing carbon allocation to nodules and improving overall plant health [28]. It was previously documented that Si (100–800 mg kg^−1^) significantly increased the soybean nodulation by 25–46% compared to the control group [19]. Abuelsod et al. reported that nanoparticles (CS-NPs) under the elevated CO_2_ conditions (645 ppm) significantly increased the nitrogen assimilation enzymatic activities (GDH, GS, and GOGAT) as compared to the control group [6]. Silicon has also been found to enhance growth, nodulation, and nitrogen fixation in leguminous plants through various mechanisms [43]. For example, Si has been shown to increase leghemoglobin formation, thereby enhancing the N_2_-fixation potential of legume root nodules [44]. It also augments the abundance of bacteroides and symbiosomes within the nodules, further promoting N_2_-fixation potential [45]. Moreover, Si promotes cell wall thickness in nodules and reduces the size of peri-bacteroid spaces, facilitating solute transport and rapid dissolving in O_2_ diffusion [45]. Additionally, Si reduces the plant requirement for lignin, a carbon-intensive compound, allowing more carbon to be allocated to bacteroid respiration and nodule organogenesis, ultimately increasing N_2_-fixation efficiency [43,45]. Recent studies have reported that SiO_2_-NPs significantly increased the module health and N_2_-fixation potential [46,47]. Collectively, these findings highlight the role of *n*-SiO_2_ in mitigating nitrogen limitations under elevated CO_2_. The observed improvements in nodule health, nitrogen fixation, and nitrogen metabolism support the use of *n*-SiO_2_ as a strategy to enhance crop productivity under future climate scenarios characterized by elevated CO_2_.

### 2.6. Synergistic Effects of Elevated CO_2_ and Nano-Silica on Soybean Uptake and Nutrient Homeostasis

**Figure 6:**
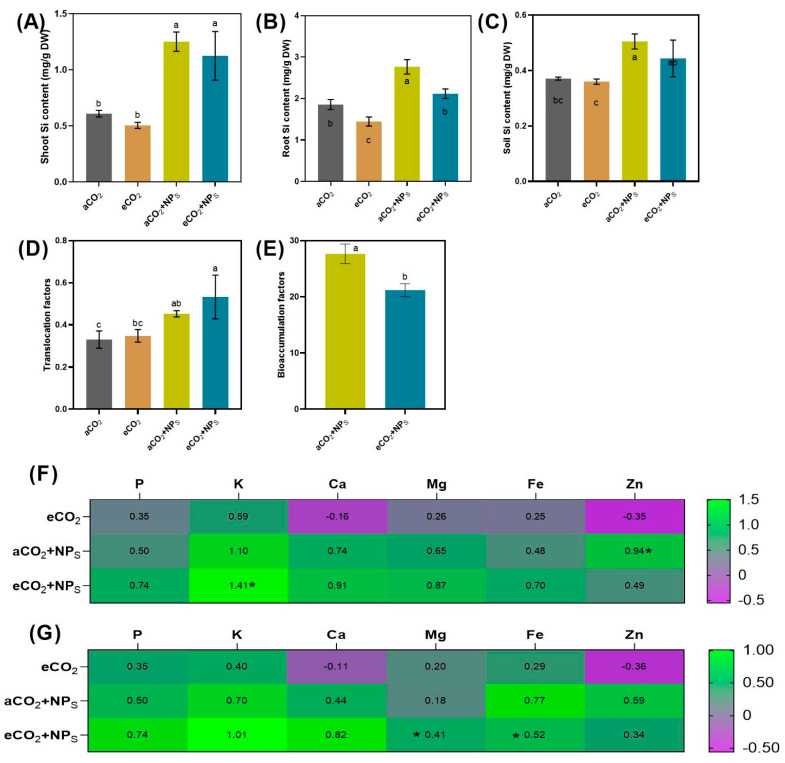
Effects of elevated CO_2_ and nano-silica on soybean uptake and nutrient dynamics. (**A**) Shoot silicon content, (**B**) root silicon content, (**C**) soil silicon content, (**D**) translocation factor, (**E**) bioconcentration factor, (**F**,**G**) heatmaps showing overall nutrient homeostasis. Bars represent mean ± standard deviation, asterisks (*) and letters indicate a significant difference between treatments.

The effects of various treatments, including aCO_2_, aCO_2_ + *n*-SiO_2_, eCO_2_, and eCO_2_ + *n*-SiO_2_, on plant Si content, translocation factor, bioconcentration factor, and elemental uptake were analyzed to understand the synergistic impacts of *n*-SiO_2_ under different CO_2_ conditions. The results revealed distinct trends and enhancements in all parameters tested, particularly when *n*-SiO_2_ was applied under elevated CO_2_ conditions. In terms of shoot silicon content (Figure 6A), both aCO_2_ + *n*-SiO_2_ and eCO_2_ + *n*-SiO_2_ treatments resulted in marked increases compared to the control (aCO_2_). Specifically, aCO_2_ + *n*-SiO_2_ exhibited a ~50% increase, while eCO_2_ + *n*-SiO_2_ showed the most substantial enhancement, with a ~100% increase, demonstrating the amplified effect of elevated CO_2_ in combination with *n*-SiO_2_ on silicon accumulation in shoots. This trend was similarly observed in root silicon content (Figure 6B), where the aCO_2_ + *n*-SiO_2_ treatment resulted in a moderate increase (~40%) compared to the control, and eCO_2_ + *n*-SiO_2_ exhibited the highest value with an ~80% increase. These findings suggest that the addition of *n*-SiO_2_ under elevated CO_2_ conditions significantly boosts the plant’s ability to accumulate Si, both in the shoots and roots. Soil silicon content (Figure 6C) also showed positive changes, although to a lesser extent. The aCO_2_ + *n*-SiO_2_ treatment led to a slight increase (~10%), while eCO_2_ + *n*-SiO_2_ resulted in a more pronounced enhancement of ~30%. This suggests that elevated CO_2_, in conjunction with *n*-SiO_2_, improves the availability of silicon in the soil, which is subsequently taken up by plants. Correspondingly, the translocation factor (Figure 6D), which measures the efficiency of Si movement from soil to plant tissues, was significantly increased in both *n*-SiO_2_ treatments. While aCO_2_ + *n*-SiO_2_ showed a moderate increase (~20%) compared to the control, eCO_2_ + *n*-SiO_2_ exhibited the highest increase (~50%), indicating that elevated CO_2_ conditions further enhance the transport of Si within the plant system. Interestingly, the bioconcentration factor (Figure 6E), which reflects the plant’s capacity to concentrate Si from the soil, was slightly decreased in the aCO_2_ + *n*-SiO_2_ treatment (~10% decrease), whereas eCO_2_ + *n*-SiO_2_ resulted in the most significant increase (~30%) compared to the control. This highlights the greater efficiency of Si accumulation in plants under elevated CO_2_ conditions when combined with *n*-SiO_2_. In addition to silicon content, the elemental content analysis (Figure 6F,G) revealed substantial upregulation of several essential nutrients under the eCO_2_ + *n*-SiO_2_ treatment. Notable increases were observed in phosphorus (P), potassium (K), calcium (Ca), magnesium (Mg), iron (Fe), and zinc (Zn), with eCO_2_ + *n*-SiO_2_ showing increases of ~74%, ~141%, ~91%, ~87%, ~70%, and ~49%, respectively, compared to the control. These results suggest that the application of *n*-SiO_2_ under elevated CO_2_ conditions not only boosts Si content but also enhances the uptake of other essential nutrients, thus contributing to improved overall plant health and growth.

Previous studies have shown that plants tightly regulate, or in some cases downregulate [48], nutrient accumulation at sub-cellular and cellular levels in a CO_2_-dependent manner in *Oryza sativa* L. [49], soybean (*Glycine max* L.), and common bean (*Phaseolus vulgaris* L.) [50], which is consistent with our current study. It has been previously observed that the presence of Si in soil enhances the availability of macro- and micronutrients to plants. Nano SiO_2_ increased root growth, along with its unique surface properties and catalytic activity, may promote nutrient retention and transport in the rhizosphere, thereby improving nutrient uptake by plants [30,51]. Xu et al., documented that SiO_2_-NPs significantly increased (~23.7%) the nutritional elements (P, K, Mn, Cu, and Zn) [18]. Previous studies have also documented that nano-SiO_2_ not only improves Si uptake capacity but also upregulates nutrient homeostasis under both normal [52] and stressed conditions [53,54]. However, we are the first to study the interactive effects of nano-SiO_2_ with elevated CO_2_ exposure. Overall, our findings consistently demonstrate that the combination of elevated CO_2_ and *n*-SiO_2_ significantly enhances silicon uptake and translocation, as well as the accumulation of key nutrients in plants.

## 3. Materials and Methods

### 3.1. Experimental Conditions and Plant Growth

The experimental soil (surface soil, 0–20 cm) was collected from the university experimental station. The air-dried soil was sieved through 2 mm mesh to remove the debris and plant residues, ensuring homogenization. The physiochemical properties of the sieved experimental soil are presented in Supplementary Table S1. We characterized the *n*-SiO_2_ following methodologies outlined in recently published studies [55,56]. Nanoscale SiO_2_ with a purity of 99.98% (17 ± 2 nm) was purchased from “Shanghai Pantian Powder Material Co., Ltd., Shanghai, China” in powder form. The characterization and sizes of the SiO_2_-NPs were analyzed using SEM and TEM (Figure S1). The size distribution and surface charge of the SiO_2_-NPs in distilled water were assessed through using dynamic light scattering (DLS) and zeta potential analysis with a Zetasizer (Nano ZS90, Malvern, UK) equipped with a He–Ne laser beam at 25 °C. The zeta-potential of used SiO_2_-NPs was −40 ± 3.7 MV, and the hydrodynamic diameter was 258.9 ± 9.87 nm. The SiO_2_-NPs were thoroughly mixed with the soil, while untreated soil served as the control (Ctrl). The treatments included soil contaminated with 100 mg kg^−1^ SiO_2_-NPs. The amended soil was left in pots for a period of two weeks to allow for stabilization. The applied SiO_2_-NPs concentrations represent realistic environmental levels.

Soybean (*Glycine max* L.) seeds (Zhonghuang-13) were purchased from the “Shouguang Seed and Seedling Co. Ltd., Shandong, China”. Uniformly sized seeds were selected and sterilized with a 3% hypochlorite sodium solution (*v*/*v*) for 10 min, then thoroughly washed with deionized (DI) water 3–4 times. The sterilized seeds were then transferred to wet filter paper and kept at 25 °C for germination. Uniform seedlings were subsequently moved into plastic pots containing SiO_2_-treated soil (100 mg kg^−1^). The experiment followed a completely randomized design (CRD), with four replicates for each treatment. Ambient CO_2_ (aCO_2_) (400 ± 15 ppm) and elevated CO_2_ (eCO_2_) (600 ± 20 ppm) levels were maintained as growth conditions. The elevated CO_2_ concentration was chosen based on projections from the IPCC-SRES B2 scenario for the year 2100 [57]. The CO_2_ for all chambers was supplied from a compressed gas tank containing liquid CO_2_. An inline fan equipped with a variable damper controlled the airflow through an external soda lime unit to regulate CO_2_ levels. The gas was supplied into the airflow of the growth chamber, and its concentration was continuously monitored using a CO_2_ analyzer (WMA-4, PP Systems, Hitchin, UK). The experiment was conducted in a climate-controlled chamber under a 16/8 h day/night photoperiod, with a photosynthetically active radiation (PAR) intensity of 350 μmol m^−2^ s^−1^, 60% relative humidity, and a constant temperature of 25 °C. Throughout the experiment, soil moisture was maintained at 60% of field capacity using DI water for on demand. The phenotypic and physiological analyses were measured after 28 days before harvesting. Four weeks after planting, plant roots and shoots were harvested, flash-frozen in liquid nitrogen, and stored at −80 °C for further analysis. The fresh and dry biomass of shoots and roots was measured.

### 3.2. Photosynthetic Pigments and Activity Assessment

Photosynthetic pigments and activity were analyzed after 27 days of exposure by measuring the fully expanded second leaf of soybean plants. Key parameters, including intercellular CO_2_ concentration (Ci), transpiration rate (Tr), net photosynthesis rate (Pn), and stomatal conductance (Gs), were evaluated using an open gas exchange system (IRGA) (LI-38 COR Biosciences, Lincoln, NE, USA). Measurements were obtained between 09:00 and 11:30 AM under standardized conditions, with a photosynthetically active radiation (PAR) of 1000 µmol m^−2^ s^−1^ and a CO_2_ molar fraction of 400 µmol mol^−1^. To ensure precision, the instrument was recalibrated before each use to maintain stable and reliable readings. Chlorophyll (*a*, *b*) and carotenoid contents were quantified by extracting pigments from the second leaf using 95% ethanol. The absorbance of the extracts was measured with a plate reader spectrophotometer (Beckman DU-640, Ramsey, MN, USA) at wavelengths of 665 nm, 649 nm, and 470 nm, respectively, following the method outlined in our previous study [52,54].

### 3.3. Biochemical Enzymes and Phytohormones Analysis

For the determination of antioxidant enzymes, 100 mg of fresh soybean leaves was ground and homogenized with a phosphorus buffer solution (50 nM, pH 7.8) at a 1:9 ratio. The homogenized samples were centrifuged at 8000–10,000 rpm for ten minutes, and the supernatants were collected for further analysis. The activities of superoxide dismutase (SOD), catalase (CAT), and along with the contents of malondialdehyde (MDA), hydrogen peroxide (H_2_O_2_), and ascorbate peroxidase (APX), were quantified using commercial kits from Nanjing Jiancheng Bioengineering Institute, Nanjing, China. Phytohormone analysis was conducted on fresh soybean shoot samples collected after 28 days. These samples were frozen in liquid nitrogen and ground into a fine powder. A 0.5 g portion of the powdered sample was then mixed with 3 mL of 80% methanol. Following centrifugation at 5000 rpm for ten minutes, the supernatant was collected. Phytohormones were quantified using enzyme-linked immunosorbent assay (ELISA), with specific polyclonal or monoclonal antibodies for each hormone and alkaline phosphatase as markers, as outlined in previous studies [58].

### 3.4. Nitrogen Assimilation Enzymes and Nitrogenase Activity

The enzymatic activities of nitrogen assimilation, including nitrite reductase (NiR), nitrate reductase (NR), glutamate synthetase (GOGAT), and glutamine synthetase (GS), were measured in fresh soybean shoots and roots according to the assay kit protocols provided by Nanjing “Jiancheng Bioengineering Co., Ltd., Nanjing, China”. Urease (UE) activity in these tissues was determined using assay kits following the manufacturer’s instructions from “Beijing Boxbio Co., Ltd., Beijing, China”. The nitrogen content in soybean shoots and roots (in dry powder form) was analyzed using an organic elemental analyzer (Vario EL model, Elemental, Germany) [52].

The nitrogenase activity in soybean nodules was evaluated using the acetylene reduction assay. Freshly harvested nodules were placed into 50 mL micro-reaction vials. Air was initially extracted from the vials using a syringe, and 10 mL of acetylene was then injected into the vials. The mixture was incubated at 30 °C for 30 min. Afterward, 500 µL of the gas mixture was sampled and analyzed for ethylene content using gas chromatography (Agilent 7890, Stevens Creek, CA, USA). Nitrogenase activity was quantified by measuring the rate of ethylene production per gram of nodule tissue [54].

### 3.5. Quantity of Mineral in Soil-Plant System

Soybean shoots and roots were freeze-dried using lyophilization at −48 °C for 72 h with a freeze-dryer (TF-FD-18S, Shanghai, China). After freeze-drying, the plant tissues were finely ground into a fine powder. A 200 mg sample of the powder was subjected to digestion with 3 mL of HNO_3_ and 0.5 mL of H_2_O_2_ in a 75 mL tube using microwave digestion (Ultra WAVE, Italy). The final volume was diluted to 50 mL with DI water and filtered through a 0.25 µm PTFE membrane. The concentrations of Si, P, K, Cu, Zn, Mn, and Fe were measured using inductively coupled plasma mass spectrometry (ICP-MS) (DRCII, PerkinElmer, and Norwalk). The mineral content in the soybean’s planted soil was analyzed through acid-wet digestion using aqua regia (3 HCl:1 HNO_3_) in a microwave digestion system. Elemental concentrations were quantified by ICP-MS using calibration standards with concentrations ranging from 0.01 to 100 ppm. Recovery rates for the analyzed elements are provided in Table S2.

### 3.6. Statistical Analysis

The experiment followed a completely randomized design (CRD) with four replicates for each treatment. The data are presented as mean ± standard deviation (SD). Statistical analysis was performed using Statistical Software (Statistix 8.1), with significance determined by one-way analysis of variance (ANOVA). Comparisons of mean values between treatments were made using the least significant difference (LSD) test. A significance level of *p* < 0.05 was considered statistically significant. Graphical representations of the data were generated using GraphPad Prism (version 8.0.2).

## 4. Conclusions

Our study demonstrates that the interactive application of *n*-SiO_2_ and eCO_2_ significantly enhances soybean growth, nitrogen fixation, and nutrient homeostasis, offering promising insights for sustainable agriculture under future climate conditions. The synergistic effects of eCO_2_ + *n*-SiO_2_ resulted in substantial improvements in photosynthetic efficiency, stomatal conductance, and carbon assimilation, leading to a 30% increase in shoot length and over 100% enhancement in chlorophyll content and photosynthetic rate. Enhanced nitrogen assimilation, evident from the upregulation of NR, NIR, GS, and GOGAT, contributed to improved biological nitrogen fixation, with nodule biomass and number increasing by 53.6% and 48.3%, respectively. Furthermore, phytohormone regulation and antioxidant defense systems were significantly boosted under eCO_2_ + *n*-SiO_2_, increasing resilience against oxidative stress while maintaining lower levels of oxidative markers. The uptake and homeostasis of essential macronutrients and micronutrients improved by 13–41% in roots and 13–17% in shoots, with Fe and Zn levels remaining stable in shoots. These findings highlight the potential role of *n*-SiO_2_ as a sustainable nano-fertilizer, capable of mitigating the physiological and biochemical challenges posed by rising CO_2_ levels while improving overall plant health and productivity. This study provides a strong foundation for future research on nano-enabled agriculture, emphasizing the potential of Si-based nanomaterials in enhancing crop performance under climate change scenarios. Integrating nano-fertilizers with elevated CO_2_ conditions could pave the way for more efficient and climate-resilient crop management strategies, ensuring global food security in a changing environment.

## Data Availability

The original contributions presented in this study are included in the article and in the Supplementary Material.

## Supplementary Materials

The following supporting information can be downloaded at: https://www.mdpi.com/article/10.3390/plants14132009/s1.
