# Supplementary material for: Silicon Nanoparticles Alter Soybean Physiology and Improve Nitrogen Fixation Potential Under Atmospheric Carbon Dioxide (CO2)
Source: Plants (Basel). 2025 Jun 30;14(13):2009. doi: 10.3390/plants14132009 (PMC12252044; doi:10.3390/plants14132009)
Supplement: Supplementary file 1 [file plants-14-02009-s001.zip › plants-3608443-supplementary.pdf]

Supplementary Information

Silicon Nanoparticles Alter Soybean Physiology and Improve Nitrogen Fixation Potential Under Atmospheric Carbon Dioxide (CO2)

Jingbo Tong*

School of Water Conservancy and Civil Engineering, Northeast Agricultural University, Harbin 150038, China

Corresponding author;
*Email: tongjingbo@neau.edu.cn

Table S1. Soil characteristics

Index	Unit	Mean value
Soil	-	Silt loam
pH	-	7.4
Available Si	mg kg^{-1}	347.19
Readily Available P	mg kg^{-1}	38.5
Rapidly available Cu	mg kg^{-1}	3.17
Organic matter	mg kg^{-1}	12.34
Rapidly available N	mg kg^{-1}	20.37
Rapidly available Fe	mg kg^{-1}	21.89

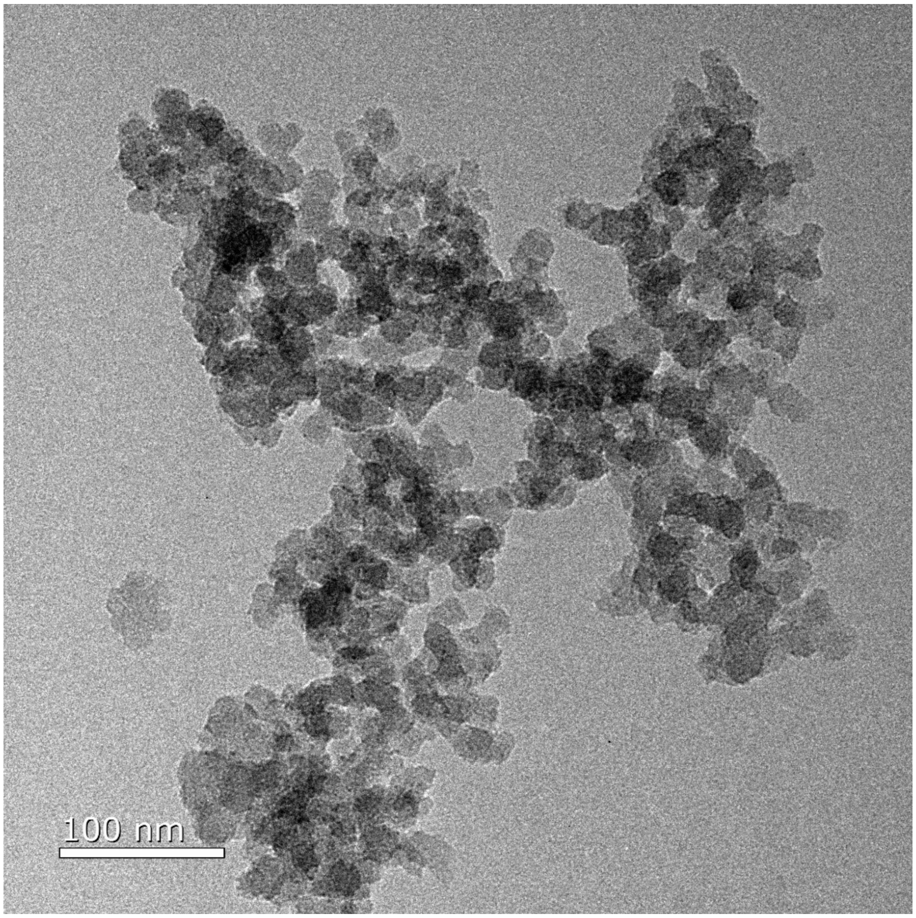

Figure S1. Transmission electron mission (TEM) image of nanoscale silicon oxide.

Table S2: List of detection, precision, and spiking recovery data for ICP-MS for the below elements.

**Elements**	**List of detection (µg L^{-1})**	**Spiking recovery (%)**	**Correlation coefficient (R^2)**	**Recovery CR M (%)**	**Correlation variance (%)**
Si	0.08	98.2	0.9999	98.3	3.91
Mg	0.064	101.6	0.9999	101.7	2.31
K	0.013	98.3	0.9999	97.9	2.71
P	0.036	98.1	0.9999	98.1	2.1
Ca	0.014	101.2	0.9999	99.3	2.23
Fe	0.063	103	0.9997	102.1	2.45
Zn	0.182	97.8	0.9997	97.6	2.89
